# Envisioning just transformations in and beyond the EU bioeconomy: inspirations from decolonial environmental justice and degrowth

**DOI:** 10.1007/s11625-022-01091-5

**Published:** 2022-01-25

**Authors:** Sabaheta Ramcilovic-Suominen

**Affiliations:** Natural Resources Institute Finland, Itäinen Pitkäkatu 4 A, 20520 Turku, Finland

**Keywords:** EU bioeconomy, Degrowth, Decoloniality, Decolonial environmental justice, Transformations

## Abstract

The European Union (EU) is adhering to decarbonization of its economy to tackle what is narrowly framed as ‘environmental issues’ of our socioecological and civilizational crises—including, but not limited to, climate change and biodiversity loss. A shift to bio-based economy (bioeconomy) is an important component of this effort. This paper applies theoretical ideas from decolonial environmental justice and degrowth, placed in the wider context of transformations, to analyse the EU bioeconomy policy within the global context, and to draw lessons and recommendations for just transformations in the EU bioeconomy policy. I identify five dominant logics and approaches in the EU bioeconomy that act as barriers for just transformations and propose alternative ones that can support such transformations. Barriers and alternatives include (1) framing ‘nature’ as a resource and service provider for humans, who are seen as separate from nature, and the need to abandon human–nature duality; (2) dominance of economic green growth and technoscientific policy solutions, and the need to place planetary justice at the centre of tackling socioecological crises; (3) a limited approach to justice, and the need to act upon climate and epistemic justice, including self-determination and self-governing authority; (4) the EU’s ambition for global leadership and competitiveness in global bioeconomic markets and governance, and the need to redefine global governance towards partnerships based on the principles of solidarity, mutual respect, reconciliation and redistribution of power and wealth; (5) hegemonic politico-economic structures and actor coalitions in charge of the EU bioeconomy, and the need for decentralized bottom-up leadership coalitions that promote direct democracy, local autonomy and sovereignty beyond state. I conclude with reflections on the politics of change and risks of co-optation, with a hope to inspire decolonial and just socioecological transformations in and beyond bioeconomy.

## Socioecological emergencies, global inequalities, the pandemic, decarbonization, bioeconomy, degrowth, environmental justice: what common threads?

The bleak findings from global assessments and literature showing the rapidly degrading state of socioecological systems continue to fill our newsfeeds. Figures and scenarios related to climate change and biodiversity loss are alarming and are among the most highlighted in policy discourses and public debates. The Intergovernmental Panel on Climate Change (IPCC) report of 2018 estimated that to have a 50–66% chance to stay within 1.5 °C of the pre-industrial level, global carbon emissions need to be reduced to around 50% from the current levels by 2030, and eliminated by 2050 (Masson-Delmotte et al. 2018). The IPCC report of 2019 shows an accelerating rate of species extinction and suggests that we are approaching a tipping point of global biodiversity beyond which a more dramatic and faster biodiversity decline is inevitable (Tucker et al. 2018). The IPCC report of 2021 echoed these messages, with higher clarity and precision than before, removing any doubts of the alarming state of the global climate, suggesting that nothing short of an immediate and complete cessation of CO_2_ and other greenhouse gas emissions can stabilise the climate (IPCC 2021). Each year is warmer than the preceding one, with the last 7 years being the hottest years ever recorded in the last 140 years (NASA Global Climate Change database). The year 2020, the hottest ever recorded, also became the turning point in history when human-made materials outweigh the living biomass (Elhacham et al. 2020). The Earth Overshoot Day, the point of time when human resource extraction exceeds the capacity for regeneration of those resources, indicates that collectively we humans require the equivalent of 1.75 planets to sustain our lifestyles (Gills and Morgan 2020). This figure is much larger in GDP-rich countries (Hickel 2020b).

These socioecological and existential crises, including climate change, are increasingly recognized as a global injustice problem due to (neo)colonial and imperial relations of extraction of labour and resources in the GDP-poor countries (many of which are in the ‘Global South’) to foster economic growth in the GDP-rich countries (‘Global North’) (Hickel 2020a, b; Hickel et al. 2021; Martinez-Alier 2020). The resulting socioecological crises have disproportionately benefited and harmed different countries and regions of the world and different segments of society (genders, ethnicities, races) (Gore and Alestig 2020; Harcourt and Nelson 2015; Hickel 2020a, b; Hickel et al. 2021; Sultana 2021). Those least responsible for the crises are, however, the most vulnerable and unprotected, importantly as a result of the continued marginalization and dispossession enabled by colonialism and the coloniality that followed it.

In the wake of the global COVID-19 pandemic, the multiple and interrelated crises that humanity is facing, even if with significant differences in urgency and severity depending on the societal groups and class (Sultana 2021), have gained renewed attention and a new dimension of interconnectedness with planetary health (Leach et al. 2021; IPBES 2020). The COVID-19 pandemic has provided momentum for questioning the current ways of doing and being in the world, and with that more focus on the viability of our current economic and environment protection systems (Büscher et al. 2021). Not only have the cavities of our global economic and health systems been exposed (Gereffi 2020) but so have also the moral cavities upon which these are based (Sultana 2021).

This increased focus relates to the concepts and initiatives for transitions and transformations, the literature on which has flourished over the last decade (Feola 2015; Feola et al. 2021; Hölscher et al. 2018). Discussions range from a need to shift our economic model towards one that prioritizes people and wellbeing over economic growth and profit (Raworth 2012) to the need for a civilizational shift and the rise of a new era (Escobar 2018; Kothari et al. 2019). Taking into account the distinction between transitions and transformations elaborated in the next section, I join the scholars who argue that transformations and transformative approaches rather than transitions and reformist approaches are needed to tackle the concurrent global socioecological and inequality crises (Gills et al. 2021; Temper 2018; Temper et al. 2018; Martin et al. 2020). Social movements have a key role to play in transformations by providing alternative ways of relating to one another and to more-than-human worlds, and by outlining alternative modes of economic and governing systems (Escobar 2015, 2018; Kothari et al. 2019; Temper et al. 2018). Degrowth and environmental justice are prominent fields of study and constitute the agenda of many social movements that call for a shift in our imaginaries, mentalities and economies, with justice at the heart of transformation processes (Akbulut et al. 2019; Gerber 2020; Martin et al. 2020, Martinez-Alier 2012).

The dominance and assumed universality of western knowledge, which justified its imposition on different hemispheres ever since the European colonial project, is part of the problem as it continues to shape development theory and practice (Escobar 2018; Shöneberg 2019). The environmental governance field represents a good example where Eurocentric ideas of environment and nature are exclusively used as a basis for designing global policy problems and solutions, dismissing indigenous knowledge systems as subjective and/or situated cultural knowledge (Biermann 2020; Rodríguez 2020). The EU has a multitude of environmental policies with direct and indirect implications for the Global South, where issues such as global deforestation, climate change and biodiversity are tackled. The EU bioeconomy policy is one of the EU policies which, even if not directly implemented or imposed on Global South countries, has direct impacts on them owing to the EU’s role in imports and trade as well as corporate investments in developing the green economy in those countries (Fuchs et al. 2020; Hickel et al. 2021; Zografos and Robins 2020).

The EU bioeconomy policy is embedded within and relies on the long-standing constellation of policy actors and institutions, similar policy problems and solutions defined by the global and EU political and economic elite (Töller et al. 2021; Vogelpohl et al. 2021). To build more ecologically sound and socially just bio-based societies and economies, the EU needs to look beyond affirmative reformist approaches that rely on the existing political and economic institutions, rules, roles, knowledge and relations, which have contributed to socioecological degradation and inequalities (Büscher et al. 2021; Gills and Morgan 2020; Martin et al. 2020; Nightingale et al. 2019; Temper et al. 2018), and seek to embrace a decolonial and pluralist approach to justice, environment and development more broadly.

The paper proceeds as follows: first I review the EU bioeconomy policy in terms of dominant bioeconomy visions and changes in the new revised bioeconomy strategy (EC 2018). Second, to emphasize the importance of global socioecological justice implications in the context of the EU bioeconomy policy, I review some of the more recent empirical findings that emphasize the socioecological costs and creation of green sacrifice zones associated with transition to green and renewable resources and energy systems. Third, I outline the theory that informs this research—decoloniality, decolonial environmental justice and degrowth—followed by main results, consisting of dominant and alternative logics and approaches that act as barriers to vis-à-vis enablers of just transformations. Finally, I reflect on the politics and the process of transformations and change.

## From a policy of green growth to a practice of green sacrifice zones

### Dominant EU bioeconomy visions and the revised EU bioeconomy strategy

In the 1970s Romanian economist Nicholas Georgescu-Roegen introduced the term ‘bioeconomics’, which directed attention to the physical, material and energy boundaries to economic growth, based on the principle of entropy described by the second law of thermodynamics (Georgescu-Roegen 1971, 2011). His ideas are considered by many as foundational for the discipline of ecological economics and degrowth alike (Asara et al. 2015; Vivien et al. 2019), even if contemporary debates on degrowth, as I will show later, draw on a host of other disciplines and therefore extend beyond Georgescu-Roegen’s notion of bioeconomics. Georgescu-Roegen’s original concept of bioeconomics and the new idea of bioeconomy launched and promoted by the EU and Organisation for Economic Co-operation and Development (OECD), despite the illusion of similarity in wording and despite the many existing visions related to the latter idea (Bugge et al. 2016; Pülzl, et al. 2014; Vivien et al. 2019), can hardly be farther apart (Vivien et al. 2019 for details).

My concern is with this “new” bioeconomy as framed in the EU bioeconomy policy. I start with the most commonly cited definition where bioeconomy is defined as “an economy where the basic building blocks for materials, chemicals and energy are derived from renewable biological resources” (McCormick and Kautto 2013). This definition is in line with the one used by the European Commission (EC), which further emphasizes the primary production sectors (such as forestry, agriculture and bioenergy), as well as services (including knowledge and innovations) as part of EU bioeconomy (EC 2018, p. 4). The focus is on bioeconomy in relation to terrestrial ecosystems, sectors and forms of land use that relate to it, including forestry, agriculture and bioenergy.

There are different visions and narratives of the EU bioeconomy (Bugge et al. 2016; Pülzl et al. 2014; Vivien et al. 2019). Importantly, however, they differ in terms of the lobbying and support they receive from stakeholders, which makes it possible to distinguish fewer dominant visions (Lühmann 2020; Korhonen et al. 2018; Vivien et al. 2019). The dominant EU bioeconomy visions and discourses are closely associated with industry, private sector, intensified biomass harvest, technological innovations, and production of novel bio-based materials. The EU dominant bioeconomy visions tend to overlook the ecological, social and cultural sustainability dimensions, which remain marginally covered (Ramcilovic-Suominen and Pülzl 2018). Consequently, the bioeconomy policy as a whole has been framed as technology-driven and biomass-based green growth bioeconomy (Bugge et al. 2016; Levidow et al. 2019), elite-driven neoliberalization of nature, which remains silent on the politics of bioeconomy (Birch et al. 2010; Levidow et al. 2019). Most recently, the policy has also been framed as a novel form of extractivism (Boyer 2019). These framings and critical analyses expose the gaps between the EU bioeconomy policies and the planetary boundaries and holistic sustainability (Gawel et al. 2019; Ramcilovic-Suominen and Pülzl 2018). I next shortly outline the evolution of EU bioeconomy policy and analyse the revised EU bioeconomy strategy (EC 2018).

On the basis of the efforts to develop biotechnology research and innovation in 1980–90s, and from there the attempts at knowledge-based bioeconomy (KBBE) during 2000–2010, the EC adopted its first bioeconomy strategy entitled “Innovating for Sustainable Growth: a Bioeconomy for Europe” in 2012 (EC 2012). While the strategy was an influential political tool that led to individual EU member states developing their own strategies (EC 2021), it faced severe criticism among the civil society and research community (for a summary of critiques, see Kurki and Ahola-Launonen 2021). Consequently, in late 2018, after a stakeholder consultation process (Lühmann 2020), the EC presented the revised bioeconomy strategy for Europe entitled “A Sustainable Bioeconomy for Europe: Strengthening the Connection Between Economy, Society and the Environment” (EC 2018).

As the name suggests, the revised strategy aims to respond to some of the critiques ascribed to the EU strategy of 2012, including the assumption of biomass sufficiency to satisfy the present level of consumption, and the socioecological damage and conflicts associated with increased production and imports of biomass (Kurki and Ahola-Launonen 2021). The revised EU bioeconomy strategy responded to these critiques in several ways. First, it diversifies the challenges at hand, which now include finite biological resources and planetary boundaries, climate change, biodiversity loss, land degradation and deforestation. Second, it highlights sustainability as the key principle, adopting the term ‘sustainable bioeconomy’. Looking beyond introductory sections and into policy measures and actions, however, sustainability is reduced to economic sustainability only, where green economic growth and green jobs have a key role. Third, locally sourced bioeconomy is encouraged, but policy measures and actions are limited to assessment, measuring, monitoring and only voluntary actions. Fourth, the solutions remain limited to technoscientific solutions, where modernised industries and green jobs for innovative production and consumption, including cascading and circular use of materials and energy, dominate. Fifth and final, the green growth and the EU global competitiveness and prosperity of its citizens are highlighted or framed as ultimate goals.

While the changes in the revised EU bioeconomy strategy are a step in the right direction, the revised strategy remains problematic in two major ways: first, the framing of challenges vis-à-vis solutions; second, the ignorance of justice and inequalities. Concerning the former, all socioecological emergencies are approached as challenges or bottlenecks that need to be addressed so that the ultimate goals (green growth, jobs, the EU’s competitiveness) may be realized, rather than something that needs to be addressed and tackled in its own right. This is evident in the closer analyses of policy measures and actions, where policy actions concerning climate change, biodiversity loss, ecosystem degradation and deforestation are limited to the need for their better understanding, assessing, measuring, and in some cases their *voluntary* addressing. On the contrary, the proposed actions concerning all aspects related to growth and the EU’s competitiveness (e.g. bio-based industry, innovation, green jobs) are elaborated more concretely and include non-voluntary policy packages. Concerning justice and equality, the revised strategy is devoid of concerns and issues related to climate justice, carbon inequality, and different carbon footprints and resource use between different sectors of society in the EU, let alone EU ecological footprint and global extraction. This is despite the growing scientific and political debates on carbon inequality and climate injustice within the EU (Gore and Alestig 2020). The reference to equality in the revised strategy is limited to “equal exploitation of bioeconomy opportunities across the EU coastal, rural and urban areas” and “equitable job creation to ensure European competitiveness” (EC 2018, p. 71). The idea of sufficiency rather than substitution alone and the need for and actions towards reducing production and consumption are also absent. The centrality of growth, the reliance on biomass and the framing of nature as a resource whose function is to serve humans and their economy remain unquestioned in the revised strategy (see also, Eversberg and Holz 2020; Kurki and Ahola-Launonen 2021).

### Displacement of socioecological costs and creation of green sacrifice zones

Global socioecological implications and justice implications are central to the EU bioeconomy for several reasons. The first concerns the EU colonial resource accumulation and contemporary vast carbon footprints (Gore and Alstig 2020; Hickel 2020a, b; UNDP 2020). The second, the lack of domestic biocapacity in most EU countries and their reliance on imports (Hoff et al. 2018; Fuchs et al. 2020; Proskurina 2018; Hickel et al. 2021). The third and final reason is the transition to bio-based, green, or low-carbon energy and economic systems, which in more than one way, and similarly to non-renewable counterparts, can and do contribute to extraction, extractivism, socioecological conflicts and various kinds of injustices. Building on Lerner’s (2010) ‘sacrifice zones’, scholars have used the concept ‘green sacrifice zones’ (Scott and Smith 2017; Zografos and Robbins 2020) to conceptualise situations where transition to green and renewable energy and economic systems leads to green grabbing, displacement, dispossession, exploitation and a range of other injustices towards marginalized groups and local indigenous and non-indigenous communities. Such sacrifice zones are reproduced in the process of developing green and renewable infrastructure (e.g. Brock et al. 2021; Dunlap and Arce 2021; Sovacool et al. 2021), as well as in the process of implementing Eurocentric ‘green’ policy solutions; the latter tend to favour scientific over local or indigenous knowledge systems, and elite groups over disadvantaged societal groups who are marginalized across various identity lines, from race to gender to class, etc. (Zografos and Robbins 2020). This leads to various forms of violence and injustices, including epistemic injustice (Harcourt and Nelson 2015; Rodríguez 2020).

Shifting to bio-based materials does not imply fewer socioecological conflicts, unless the sources of environmental and social injustices, such as extractive mentalities, exploitive human–nature relations, economic and cultural domination and power asymmetries are addressed. An emerging body of literature documents the harms and conflicts over extraction of resources needed for decarbonization of our economies and for growth of green and bio-economies (Huff and Orengo 2020; Temper et al. 2020; Zografos and Robins 2020), which in some cases have led to murder of those resisting extraction (Butt et al. 2019; Le Tran et al. 2020; Menton and Le Billon 2021).

Some of the most serious examples of violence and extraction are associated with biomass for production of biofuels (Bastos-Lima and Gupta 2014; Backhouse et al. 2021), hydropower (Del Bene et al. 2018) and wind energy (Dunlap and Arce 2021), which lead to food insecurity, forced displacement, loss of livelihoods, oppression, dispossession and loss of rights and access to land and resources. Temper et al. (2020) show that globally conflicts associated with biomass and hydropower extraction are comparable to those of extraction of non-renewables. Yet, the political momentum of decarbonization, bioeconomy and transition to green energy have helped reframe energy sources such as biomass and hydropower as ‘green’ (Zarfl et al. 2014). Wind energy is argued to produce ‘infrastructural violence’, where communities are relocated or otherwise affected by heavy infrastructure (Dunlap and Arce 2021). When locals resist the land grabs and dispossession this has led to physical violence, intimidation and in the worse cases death of activists (Butt et al. 2019; Menton and Le Billon 2021). Local resistance movements have nonetheless been effective in slowing down extractive projects worldwide (Temper et al. 2020). While conflicts and violence tend to be more severe in cases where indigenous and politically and socially marginalized groups are involved (Butt et al. 2019; Menton and Le Billon 2021), they occur across different geographies, including in EU countries (Brock et al. 2021; Sovacool et al. 2021).

This short overview reveals the political nature of bioeconomy and decarbonization projects, where issues of justice and power and creation of winners and losers appear to be inevitable. Considering that some of the key actors in the bioeconomy and in GDP-rich EU member states are agribusinesses and the pharmaceutical industry (e.g. Korhonen et al. 2018), the risks and concerns only increase (FFRC 2019). Considering the path dependency, power relations and institutions embedded in the global environmental governance, it is highly unlikely that the local dispossession and violence associated with many of such policies (Huff and Orengo 2020; Milne and Mahanty 2019; Ramcilovic-Suominen et al. 2021) are to subside in the case of bioeconomy. On the contrary, given the importance of market-based and technoscientific solutions in the EU bioeconomy, the same actor coalitions and the path dependencies (Vogelpohl et al. 2021), there are numerous reasons to be concerned about the impacts of the EU bioeconomy policy in the Global South and global peripheries elsewhere (Sovacool 2021; Sovacool et al. 2021; Zografos and Robins 2020).

## Decoloniality, decolonial environmental justice and degrowth as tenants of transformations

### Transformations

While the literature on transformations has flourished and different strands have emerged (Feola 2015), some common trends can be observed. Those include (1) reference to the Anthropocene and planetary boundaries (Gills and Morgan 2020; Feola 2015; Steffen et al. 2007) and (2) need for fundamental shift, as opposed to minor or incremental changes (Feola 2015) and (3) radical and widespread societal changes as opposed to changes in a certain sector or policy domain (Holscher et al. 2018). Beyond these generic features, there is little agreement about what transformation is, what ends it should achieve, or how (Bluwstein 2021; Gram-Hanssen et al. 2021). The uncertainty about the *what* and the *how* of transformations, I argue, is part of the definition of transformations as an uncertain, emergent, heterogenic and multifaceted process which is not managed and instituted under the existing structures, forms of knowledge and definitions. This again sets transformations aside from the more structured and managed processes of transitions that are expected to lead societies towards known and desired ends and futures (Holscher et al. 2018; Pelenc et al. 2019; Temper et al. 2018).

I highlight in particular the politics or power dimensions (Martinez-Alier et al. 2016; Temper et al. 2018; Scoones 2016) and justice dimensions (Martin et al. 2020; Temper et al. 2018) as central features of what Temper et al. (2018, p. 5) term ‘radical transformations’. The word “radical”, originating from the Latin* radix* meaning roots (Pugh 2009), in this context means changing the root causes, as opposed to symptoms of a problem. Radical transformations therefore focus on addressing the underlying structural root causes that (re)produce ecological destruction and societal injustices, many of which boil down to the politics, power, intersectionality, and the multiple justice dimensions. The power/politics and justice dimensions, as central elements of transformations, are mutually connected and in practice reproduce one another. For instance, power configurations such as capitalism, patriarchy, and state reproduce injustices against certain sections of society and identities defined through gender, race, ethnicity or caste (Le Tran et al. 2020; Kojola and Pellow 2020; Sakshi 2021; Sultana 2021). From the plethora of fields offering visions concerning transformations, I focus on decolonial environmental justice and degrowth.

### Decoloniality

Decoloniality is theorized in different contexts and approached from different perspectives. There is a general agreement, however, that decoloniality represents a critique of modernity (Quijano 2000, 2007), and of modern notions of development, which reproduce various forms of violence and oppression, including extractivism, racism, patriarchy, and cultural domination (Maldonado-Torres 2007; Lugones 2010). Building on Fanon’s decoloniality of mind (Fanon 1967) various scholars have focused on decoloniality of imaginary and being (Latouche 2015; Maldonado-Torres 2007; Mignolo and Walsh 2018). Decoloniality is also discussed in the context of human-nature duality (Rodríguez 2020; Temper 2018), power relations and forms of knowledge (Santos 2008). Unlike decoloniality of mind perspectives, indigenous perspectives frame decoloniality in more material and concrete terms and aims, which include ending structural systemic oppression and violence of colonialism, as well as repatriation or restitution of indigenous land (Tuck and Young 2012).

Hence, an important scholarly debate on decoloniality concerns the questions of what is to be decolonized (i.e. the object of decolonialisation) and whether decoloniality should be used as a means to address a range of socio-political issues (i.e. decoloniality as a means), or an end in itself (i.e. decoloniality as an end) have emerged among decolonial, postcolonial and degrowth scholars (see for example D’Alisa et al. 2015; Deschner and Hust 2018; Feola 2019; Garcia-Arias and Schöneberg 2021; Tuck and Yang 2012). Concerning the object of decoloniality, as noted above, the debate revolves around decoloniality of material (i.e. land and colonial structures of domination), and of immaterial, including mind and imaginary (i.e. knowledges, worldviews, significations). Decoloniality of mind, originally introduced by Fanon (1965, 1967), has since been used by many decolonial and some postcolonial and degrowth scholars. For example, Latouche (2015) frames decoloniality of imaginary as decolonizing of culture, meanings, and significations. Even if he discusses decolonizing imaginary directly in relation to a range of societal, structural and political issues, arguing especially against economic growth, some degrowth scholars criticize Latouche for engaging only slightly with the questions of power and conflicts (Feola 2019). Decolonial and post-colonial scholars, in contrast, problematize decoloniality of imaginary as defined by Latouche as an appropriation of the term that takes decoloniality away from its core and intended objectives, which should be repatriation of indigenous land and life (Deschner and Hurst 2018; Tuck and Yang 2012). Similarly, some scholars argue for a use of decoloniality strictly in relation to undoing colonial histories and injustices (decoloniality as an end in itself), while others approach it in the context of, and for addressing a range of socio-political issues, including justice and education for example (decoloniality as a means).

Acknowledging that tensions between the different positions on the object of decoloniality can emerge, I am inclined to suggest that the two are not necessarily mutually exclusive. In fact, decolonizing the immaterial, that is the mind and imaginary, can act as a precondition for decolonizing the material, since to decolonize land and structures of colonial oppression requires, decolonizing the imaginary and mind is necessary. Such synergies are, however, less certain when it comes to framing decoloniality as a means (i.e. using decoloniality for and in the context of tackling various social and ecological issues) versus framing it as an end (i.e. decoloniality as undoing the colonially rooted systemic oppression and the aim of repatriation and restitution of indigenous lands). While the former may not directly obstruct the latter, it does not by default support it either. As Tuck and Young (2012) convincingly argue, using decoloniality argument for advancing various societal and political agendas and aims—regardless how benevolent they might be—can be harmful as it shifts the attention from the key point and aim, that is ending the colonial oppression and violence and repatriation of indigenous lands. In this context, the importance of remaining vigilant to numerous attempts to co-opt and reduce the radical ideas and political agendas of decoloniality cannot be overstated. Strengthening alliances among decolonial, postcolonial and degrowth scholars and activists is an important step towards the right direction, providing that dismantling the system of cultural and political violence of colonialism is an important part of their political projects and ideas.

### Decolonial environmental justice (DEJ)

Decolonial environmental justice (DEJ) draws on the ideas of decoloniality and emphasizes the need to decolonize concepts, meanings and epistemologies embedded within the environmental justice literature (Menton et al. 2020; Ramcilovic-Suominen et al. 2021). DEJ emerges as a response to critiquing “mainstream” environmental justice (EJ) (Menton et al. 2020), which proposes three and sometimes four dimensions of justice, including participation, distribution, recognition (Fraser 1998; Schlosberg 2007) and capabilities (Nussbaum 2011). The critiquing of the EJ framework refers to its western or Eurocentric worldviews, imaginaries and politics, which do not fit those in many countries in the Global South where Eurocentric EJ is commonly applied (Alvarez and Coolseat 2018; Rodríguez 2020; Temper 2018).

Seminal works on DEJ (Alvarez and Coolseat 2018; Rodríguez and Inturias 2018; Rodríguez 2020; Temper 2018) highlight three central aspects. The first concerns the need to move *beyond human–nature dichotomy*. This aspect builds on indigenous cosmologies (i.e. theories of the world), but is increasingly used as a framework for rethinking the human place and relations to other forms of life in other contexts (Biermann 2020; Gebara 2021). In this view, humans are an intrinsic and embedded part of the natural world and the underlying cause for socioecological crises relates to humans dominating nature, rather than living *with* nature. The notion of distributional justice is challenged because the very idea of distributing nature assumes that humans are separate from nature and can distribute it more equally between themselves (Temper 2018). Imposing distributional justice is a form of epistemic injustice to indigenous and non-indigenous local groups, given that in so doing their worldviews and epistemologies are ignored and dominant Eurocentric ones imposed (Rodríguez 2020). DEJ calls for a breaking of the human–nature dichotomy, which degrowth scholars term reciprocal and regenerative human–nature relationalities (Hickel 2020a).

The second aspect, *epistemic justice and the right to self-determination*, concerns the imposition and domination of one particular way of knowing the world at the expense of other ways, which is termed as epistemic domination or violence. ‘Speaking for others’ and assuming that other knowledge systems are subordinate and therefore need to adopt the ‘supreme’ western Eurocentric one creates cultural and political oppression and slow violence (Tuhiwai Smith 1999). Temper (2018) argues that recognitional justice (Fraser 2000) can lead to inclusion of other onto-epistemological orientations within the dominant colonizing ones. Consequently, DEJ requires going beyond recognition and towards cultural and political self-determination, where people rely on their own sociocultural and political norms and structures (Rodríguez 2020; Temper 2018).

The third aspect, *self-governance and self-governing authority*, while tightly linked to the other two, concerns the procedural EJ element (i.e. participation). The argument is for the need to go beyond participation and towards the self-governing authority of informal indigenous or other customary structures and authorities, which exist in parallel with formal state systems (Lund 2011). The implication is that indigenous and other traditional authorities have the right to govern their own territories within their own indigenous and/or customary governing structures. This aspect is opposed to procedural justice which argues for participation *within* the existing domineering political and legal structures and which can lead to cultural and political assimilation (Sakshi 2021; Rodríguez 2020).

### Degrowth

While there are different perspectives on degrowth, there are also some well-agreed key propositions, including that degrowth is not simply about descaling the economy and current practices but about changing them altogether, together with their underlying logics (Asara et al. 2015). Hence defining degrowth as a society with a smaller metabolism (energy and material throughput an economy) only portrays one side of the coin (Kallis et al. 2020). Degrowth signifies a society with different structures that serve different functions, needs and relations. The emphasis is on a good life, care, wellbeing, community and living well with less for all, implying redistribution and more equal sharing of resources globally (O’Neill 2020; Raworth 2012). The relevance of direct democracy and local autonomy is central (Parrique 2019) as is the need to define our limits autonomously, collectively, and democratically (Kallis 2013). Finally, the degrowth perspective also calls for radical change in infrastructure, technologies and monetary system, with the aim of strengthening sharing and communing, direct democracy, wealth and power redistribution (Kallis et al. 2020), and of paving the way for more democratic and decentralised technologies and financial systems (Douthwaite 2012; Pensera and Fressoli 2019).

Degrowth builds on critiquing growth-oriented paradigms, including green growth, green economy and sustainable development (D’Alisa et al. 2015; Kallis et al. 2020). Degrowth scholars argue that such frameworks and paradigms have been co-opted and reoriented from their original ideas, towards serving economic growth at the cost of nature or environment, society and cultures (D’Alisa et al. 2015). An important feature with direct implications for the policies such as the EU bioeconomy is the degrowth’s focus on sufficiency (rather than substitution, for example) and affinity for voluntarily limiting our consumption and accumulation (Kallis 2013). Degrowth’s focus on global environmental justice, care and reciprocity, which are increasingly articulated in the literature through the feminist and decolonial perspectives on degrowth (Dengler and Seebacher 2019; Mehta and Harcourt 2021; Nirmal and Roscheleau 2019; Singh 2019), is also vital to EU bioeconomy. Kallis et al. (2020) outline a number of strategies which they argue offer alternatives to the current capitalist extractivist economy. These strategies include carbon and wealth taxes, debt cancelation of the former colonies and GDP-poorer countries, redistribution of economic and political power across countries and regions of the world and scaling down environmentally polluting sectors and industries while promoting locally sourced production and consumption economies. While these measures are not exhaustive, especially when it comes to onto-epistemological aspects and decolonial justice, they hold tremendous potential for informing and inspiring EU bioeconomy policy to embrace socially and ecologically just approaches in building European bio-based societies.

## Drawing on DEJ and degrowth to identify barriers to and alternatives for socioecological justice and transformations

Applying the theoretical framework outlined above to the EU bioeconomy policy described in “From a policy of green growth to a practice of green sacrifice zones”, I first identify the dominant approaches and logics in the EU bioeconomy that act as barriers to socioecological justice and transformations, and then propose alternative ways and logics to support just transformations within the EU bioeconomy in its global context. More attention is given to the alternatives, rather than the better-known barriers and problems. The barriers include:

(i) Framing the web of life (nature) as a resource and service provider for humans, which is based on and strengthens extractive and ‘rent-seeking’ behaviour and relations to nature and at best promotes market-friendly nature-based solutions, such as carbon sequestration and trading. Responding to this framing calls for reimagining the dominant Eurocentric human–nature relationality and for abandoning human–nature and other forms of duality (Biermann 2020; Gebara 2021; Hickel 2020a; Temper 2018). Relationality concerns the broader way we humans view, live and relate to the world around us. It refers to an idea that humanity is embedded and relationally constituted within it (Gebara 2021, p. 238).
(ii) Dominance of green growth logic and technoscientific policy solutions that promote large-scale economic and industrial activities at the expense of socioecological burdens and small-scale technologies and activities. This approach does not question the current production and consumption levels and prioritizes industrial development that comes at the expense of small-scale, community-based and local economic activities, and the sociocultural and socioecological sustainability (O'Neill 2020; Kallis et al. 2020).
(iii) A limited approach to socioecological justice, where only procedural and distributive justice elements are visible, but limited to key stakeholder participation (i.e. European businesses, industry, policymakers and research institutions) and to equitable distribution of job opportunities. Global socioecological injustices, historical, colonial and current exploitation and inequalities related to climate change responsibility and ecological footprints between different societal groups and countries are ignored in the EU bioeconomy.
(iv) EU global leadership and competitiveness in globalized bioeconomic markets as a goal, which motivates relocation of socioecological burdens of EU bioeconomy to its domestic and global peripheries. This furthers reproduction of neo-imperial and neo-colonial relations with the countries in the Global South in the name of European competitiveness, development and carbon-neural economy.
(v) Hegemonic politico-economic structures and actor coalitions in charge of EU bioeconomy, including international economic organizations, such as OECD, and transnational businesses. These actor coalitions and their policy initiatives and decisions cooperate with state structures, overlooking the legal plurality and indigenous structures as well as state coercion and violence. Recentralization and state-centred initiatives are reproduced in the countries where international policies are implemented, which can lead to further marginalization and violence (Harcourt and Nelson 2015; Ramcilovic-Suominen et al. 2021).

The proposed alternatives are intended to strengthen justice and plurality and to discourage discrimination against and violations of other knowledge, practices, and actor constellations. These barriers and alternatives are organized in three groups, as shown in Table 1.

**Table 1:** Barriers to just transformations and alternatives: the case of EU bioeconomy with its global implications

Barriers to just transformations in the EU bioeconomy with global implications	Alternative ways and logics to support just transformations within the EU bioeconomy
*Barriers and alternatives concerning relationalities and ways of being*	
1. Framing of nature as a resource and service provider for humans	1. Beyond human–nature dualism and extractive relations towards reciprocal and regenerative relationality
*Barriers and alternatives most specifically linked to bioeconomy*	
2. Dominance of (green) growth and technoscientific policy solutions that favour industrial activities at the expense of socioecological burdens	2. Beyond the logic of growth and technoscientific solutions towards institutionally supported, sufficiency-oriented lifestyles and means of production that respect planetary limits and planetary justice
3. Limited approach to socioecological justice, where procedural justice is limited to “key stakeholder participation” and distributive justice is limited to equitable distribution of economic opportunities	3. Beyond stakeholder participation and equitable distribution of economic opportunities towards decoloniality, plurality, climate and epistemic justice, and full recognition for cultural and political self-determination and self-governing authorities
*Barriers and alternatives that concern the politics of change*	
4. EU global leadership and competitiveness in globalized bioeconomic markets as a goal in itself	4. Beyond global competitiveness and harmful competition towards partnerships based on principles of local autonomy, solidarity, mutual respect, reconciliation and redistribution of power and wealth
5. Hegemonic politico-economic structures and actor coalitions in charge of EU bioeconomy	5. Beyond dominant actor coalitions, top-down initiatives and state coercion towards decentralized bottom-up leadership that promotes direct democracy, local autonomy and sovereignty

### Alternative ways and logics to support just transformations within the EU bioeconomy

In what follows we present the alternative approaches to the identified barriers, elaborating also on the barriers in further detail.

(i) Beyond human–nature dualism and extractive relations towards reciprocal and regenerative relationalities with more-than-human ‘natures’

“Bio-based products go far beyond biomass processing. They capitalize on the unprecedented advances in life sciences and biotechnology… that, coupled with the digital revolution, allow [one] to use nature’s “biological assets” (its biochemicals and biomaterials) and its “biomimetic assets” (its functions and processes) to generate significant new sources of economic value and future revenue” (EC 2018, p. 42). This citation from the revised EU bioeconomy strategy is a common example of human–nature duality where humans and nature are seen as separate from one another, with the former dominating the latter.

Human–nature dualism and the associated extractivist logics position human species as supreme to other species and forms of life, which justifies human domination, extraction, and exploitation of other forms of life for the benefit of humans. Human–nature dualism was consolidated in the process of European enlightenment and was instrumental in justifying European colonialism, dispossession and enslaving of colonized non-Europeans and capitalist endeavours that began some 500 years ago (Schöneberg 2019; Hickel 2020a). This duality persists and helps maintain domination of humans over more-than-humans (animal and plant species), reducing them to mere resources and/or service providers to one supreme species.^1^ For instance, concepts such as ‘ecosystem services’ effectively reproduce such logics (Sullivan 2010).

Facing unprecedented socioecological breakdown, the time is ripe for Europe to re-examine its epistemologies (knowledge systems) and ontologies (philosophies of life) concerning human relations with the wider ecology. Indigenous cosmologies or philosophies of life are known for positioning all forms of life—animals, plants, trees, bacteria, viruses—as part of one whole biosphere or ecology (Gebara 2021; Gram-Hanssen et al. 2021). Beyond indigenous cosmologies, modern and western science and scholars also increasingly question the human–nature duality, stressing the importance of epistemic plurality (Biermann 2020; Pascual et al. 2021).

Once we see ourselves as interconnected, embedded in, and dependent on other forms of life and species, and accept the sentiency of other forms of life (i.e. that they are morally equal to us), not only our relations with them but also our policy goals are more likely to shift from dominance, extraction and competition to those of care, respect, reciprocity, responsibility and nourishment (Hickel 2020a, b). But how do we get there? My argument is not for the EU bioeconomy and EU societies to adopt animism, but for human–nature narrowly framed relations to be re-examined (Gebara 2021). I also do not suggest that European or any other society ceases to use other forms of life, as this contradicts the (co)existence and the very existence of societies in question. Besides, examples from Latin America, where the neoliberal grab and commodification of nature have continued despite indigenous ontologies being recognized and given space in the countries’ laws and constitutions, suggest the need for radical change in practice as well. Yet, I argue that rethinking the ways we relate and interact with and use other forms of life, by embracing reciprocal relationality, is the first and necessary step to radical change. Reciprocity here implies taking not more than you need and returning the favour. Returning can be done by regeneration or replenishing the forms of life that we interact with (e.g. forest, land), which ensures continuity of their regenerative capacities; an example would be regenerative agroecological practices (Hickel 2020a). Any efforts aiming to support a transition to a post-fossil economy that can be sustained in the long run, as the EU bioeconomy strategy and other similar policies claim to intend to, must go beyond colonising, exploiting and extracting other forms of life. Such attempts and policies need to urgently rethink and abolish human–nature duality, promote reciprocal relationality between humans and other species and forms of life.

(ii) Beyond green growth and technoscientific solutions, towards institutionally supported, sufficiency-oriented lifestyles and means of production that respect and prioritize planetary limits and planetary justice

The EU bioeconomy recognizes the degraded states of life cycles and proposes a circularity of energy and materials and a decoupling of economic growth from use and degradation of ‘nature’. Both circularity and decoupling are vital, but unattainable at the rate and within the timeframe needed. The scale of technological readiness and particularly the level of economic and political readiness lag significantly behind the urgency of socioecological crises, climate change and biodiversity loss (Hickel and Kallis 2019; Parrique et al. 2019). Regarding circularity, only a portion of the materials and energy sources that we use can be recycled, as a large share of primary materials is degraded through use and recycling itself is energy intense (Giampietro 2019; Parrique et al. 2019).

Hence there is a need for strategies which focus on living within planetary boundaries and planetary justice as a precondition for curbing CO_2_ emissions and inequalities. One such strategy is the principle of *sufficiency and voluntary frugality* (Kallis 2013). Sufficiency is opposed to excess and accumulation and can be explained using Raworth’s (2012) idea of ‘living within the doughnut’ or living within a safe and just space for humanity, where everyone has a solid social foundation without overshooting the ecological ceiling or limits. Embracing sufficiency in GDP-richer EU countries can lead to faster reductions of throughput of energy. Since the wealth accumulation by GDP-richer countries and economic elites relocates social and ecological damage to global peripheries, adopting sufficiency in the GDP-rich countries also directly advances global socioecological justice.

Supporting local(ized) production and consumption of resources and services is also important because such local and small-scale activities perform better ecologically owing to their shorter value chains. Being embedded within sociocultural and socioecological systems, local(ized) systems of economic exchange are potentially more sustaining and regenerating of socioecological systems (Parrique 2019), and for the same reason of place embeddedness, they are also likely to be more democratic (Kallis et al. 2020; Parrique 2019). However, such local initiatives require policy and institutional support that a bioeconomy policy can provide. Such support includes, for example, reforming and democratising the monetary system and tax relief to support public goods, local producers and livelihoods (Douthwaite 2012; Mellor 2017). Examples of alternative and local initiatives and cooperatives for providing collective goods and services exist throughout Europe (Kallis et al. 2020) and the world (Kothari et al. 2019). Promotion and support of cooperatives and community-led projects for provision of food, clothes, cosmetics, shared transportation and housing, and renewable energy is needed in the EU bioeconomy policies and discourses as a complement to the narrow focus on innovation and technologies. This in turn implies more self-reliance and local autonomy to decide, and less dependence on private and industry-led provision of new materials and technologies.

(iii) Beyond stakeholder participation and equitable distribution of economic opportunities, towards climate and epistemic justice, local autonomy, self-determination, and self-governing authority

As discussed in “From a policy of green growth to a practice of green sacrifice zones”, the EU bioeconomy policy adopts a rather narrow focus when it comes to procedural and distributive justice that is limited to participation of stakeholders and distribution of economic opportunities. Recognitional justice, which deals with marginalization and cultural identities (Fraser 1998, 2000), is the least reflected in the bioeconomy policy. This may partly be because it is seen to be part of other Directorate-Generals (DG) and their policies and regulations (e.g. DG for justice and consumers). Proposing a decolonial justice approach is crucial if the EU is to ensure plurality and justice and to address inequalities, both globally and domestically (Schöneberg 2019).

At a global level, it is crucial that the EU countries and the block as a whole acknowledge and address the injustices associated with the historic colonial past, as well as with the contemporary and non-proportional burdens and injustices associated with its high living standards, and its environmental, nature protection and development policies more broadly. This is necessary for starting to imagine a decolonial turn in the EU development approach and policies. This also concerns and requires a shift from policies that rely on technoscientific and market-based solutions and which go beyond the rhetoric of participation and selling ‘good governance’ packages to ‘developing countries’. Taking a decolonial turn means (1) embracing and acting upon climate and epistemic justice, (2) embracing plurality of knowledge and epistemologies, and (3) recognising local autonomy of indigenous people, their cultural and political self-determination and self-governing authority, their territorial rights and practices of living with and within the web of life (Kothari et al. 2019; Temper, 2018).

(iv) Beyond global competitiveness and harmful competition, towards partnerships based on the principles of local autonomy, solidarity, mutual respect, reconciliation and redistribution of power and wealth

The EU bioeconomy, like the green economy to which it is closely associated, is embedded within the global trade, power and economic relations, which are in turn embedded within colonial and neo-colonial legacies of racially driven domination, extraction of labour and ‘natural resources’ and exploitation and dispossession of people in the Global South (Harcourt and Nelson 2015; Hickel 2020a, b). If the EU leadership is to respond to growing pressure including from its own especially younger and increasingly protesting citizens and civil society organizations, demanding climate justice and an end to fossil capitalism, it is not the global competitiveness the EU should be concerned about. Rather efforts should be made towards recognition of responsibilities and commitment to reparations for the wrongdoing of the past and change of the current trends and politics. Yet not only is there a shortage of such commitments and change but policies like the EU bioeconomy signals continuity and escalation of neo-colonial domination and plunder in the Global South (Fuchs et al. 2020; Hickel et al. 2021; Zografos and Robbins 2020). It is not sufficient to promote use of “domestically available sustainable renewable resources” if they are not defined or elaborated upon (Kurki and Ahola-Launonen 2021). To avoid reproduction of green sacrifice zones (Zografos and Robbins 2020), EU policies with relations to and implications for the Global South need to change course radically, and here are just a few suggestions: (1) reinvent EU relations with and role in the world, with the humility of a former coloniser, where responsibilities for historical/colonial and modern/neo-colonial debt, burdens and injustices are acknowledged and acted upon; (2) reform EU policy and institutional framework with an aim to fostering reconciliation, reciprocity, respect, care and solidarity with the Global South and marginalized groups across the peripheries, as opposed to exploitation and violence; (3) abandon the ‘imperial mode of living’ by tackling non-proportional and wasteful consumption and extraction and thus alleviate pressure on lands, peoples, and ‘natures’ at home and elsewhere; (4) implement reforms for redistribution of wealth and power in a way that addresses current inequalities and repairs historic, as well as contemporary injustices and violence associated with the EU corporate extraction and dispossession; (5) implement policy reforms that allow the EU to operate in full recognition of domestic and, where relevant, local indigenous and customary territorial rules and rights, including cultural and political self-determination and self-governing authority. Relying on the state as the only legitimate actor tends to reinforce marginalization and violence against indigenous and/or rebellious groups (Dunlap 2021a; Pellow 2018; Ramcilovic-Suominen et al. 2021).

(v) Beyond dominant actor coalitions, top-down initiatives and state coercion towards decentralised bottom-up leadership that promotes direct democracy, local autonomy and sovereignty

Owing to the European colonial project, spaces and territories in the Global South remain characterized by parallel existence of formal/state and informal/customary structures, which compete for power and authority (Lund 2011). This remains one of the major causes for conflicting relations between the state and sections of society that do not always recognize the state authority but rely on traditional norms and political authority. Contrary to this reality, the EU institutions and global governance organizations (World Trade Organization, World Bank, International Monetary Fund, OECD) collaborate exclusively with state organs, while ignoring the evidence that the state does not always represent all citizens equally (Kojola and Pellow 2020; Sakshi 2021) and plays an important part in oppression, violence and coercion (Dunlap 2021a). This leads to consolidation of state control of people and territories that previously operated more independently, fostering thus state-sponsored dispossession and violence (Ramcilovic-Suominen et al. 2021; Menton and Le Billon 2021).

Systemic and structural shifts in global governance structures, discourses and power relations are required to allow for direct representation of indigenous people and other marginalized groups, by their own elected authorities, norms and structures, including direct democracy. There is a need to decentralize power and authority and encourage bottom-up leadership for transformations that can offer a safe political space for marginalized or oppressed groups (Stephens 2020). Local and bottom-up leadership examples across the world show an affinity for direct democracy, local autonomy and non-violence (Kothari et al. 2019). Examples of people-centred bottom-up leadership where people come together to organize and govern their political, economic and ecological affairs exist across the world. Such leaderships challenge state oppression, racism, casteism, sexism, patriarchy and other forms of social injustice and marginalization, including those rooted in their own culture (e.g. tackling discrimination related to gender, ethnicities, caste etc.). Examples include, but are not limited to, the Kurdish Rojava women’s movement for autonomy and direct democracy (Gills and Hosseini 2021) and various movements for ecological stewardship, food and seed sovereignty, economies of caring and sharing, self-determination and autonomy (Kothari et al. 2019).

Progressive political leadership in the EU and the USA alike needs to embed itself within the anti-racist, feminist political leadership to a greater extent as to defend their rhetoric and commitment to social justice, security, race and housing (Stephens 2020). We can see signs of hope on the streets, where youth protesting for new climate policies have had significant impacts.

## Transformations, co-optation, and politics of change: EU bioeconomy and beyond

The EU bioeconomy policy primarily aims to advance economic sustainability, green growth, and EU global competitiveness, by substituting non-renewables for renewables, modernizing production and consumption, boosting technology, research and innovation, and promoting circular and cascade use of materials and products. Socioecological emergencies and existential crises, such as climate change, biodiversity loss, deforestation and ecosystem degradation, are framed as challenges to achieving these goals. Based on the decolonial environmental justice and degrowth perspectives, the agenda proposed in the bioeconomy policy and the revised strategy is insufficient in several ways. The EU’s ecological and climate debt are left unaddressed. Global inequalities and responsibilities for the EU’s historical colonial and current neo-colonial extraction and appropriation of resources from the Global South are sidelined. Sufficiency or decreases of EU production and consumption levels, which fuel domestic and global socioecological burden and injustices, are not among suggested policy measures. Economic sustainability, creation of ‘green jobs’, circular economy approaches and the focus on participation of stakeholders and equal economic opportunity within Europe are insufficient to tackle the socioecological crises at hand. The same holds for substitution of non-renewables with renewables, coupled with technology, research and innovation, and EU global competitiveness.

A significant part of this paper deals with the need to go beyond certain approaches and logics, identified as the key barriers to just transformations in the EU bioeconomy, and with alternative approaches and logics to facilitate such transformations (“Drawing on DEJ and degrowth to identify barriers to and alternatives for socioecological justice and transformations”). Rather than proposing far-fetched policy recommendations for more transformational and more just politics in the EU bioeconomy policy domain, in what follows I reflect upon the process of change and risks of co-optation, with a hope to inspire decolonial and just socioecological transformations in and beyond bioeconomy.

The concepts and processes of decoloniality, degrowth and transformations are crucial for the radical changes, as argued above. Yet, each of these concepts and political agendas is highly susceptible to co-optation that leads to dilution of their radical ideas, followed by their absorption into the ‘ordinary’. This co-optation can, and usually happens from the outside—by actors who favour status quo (Coy 2013; Katz-Rosene and Paterson 2018; Stephens 2020). Co-optation can also happen from the inside, and by the proponents themselves, when faced with a temptation for proliferating and mainstreaming their concepts and political agendas. While co-optation from the outside is the most dominant across all three concepts and political agendas, it currently appears to be the most extreme in the case of transformations, where the term is used to describe and therefore legitimize sociotechnical and eco-modernist solutions as ‘transformative’ (Kruus and Hakala 2017; Tome Valencia Hamilton and Ramcilovic-Suominen forthcoming; UNECE 2012). Decoloniality, inter alia, appears to be prone to co-optation by often well-intended group of actors, including scholars, who may use the term without properly engaging the theory and previously published literature, and may end up reducing the term to a buzzword or a metaphor (Deschner and Hust 2018; Tuck and Yang 2012). Internal co-optation is highlighted as a potential risk to degrowth. As degrowth ideas start to gain wider popularity, including among policymakers, they tend to reduce degrowth to a toolbox for “broadening the sustainability debate (and for meeting EU’s) sustainability ambitions” (EEA 2021; p. 2), largely omitting the sociocultural and justice dimensions of degrowth. Some scholars have noted that degrowth proponents in some cases flirt with policy approaches that can be considered rather reformist, statist, and non-heterodox, risking internal co-optation (Deschner and Hust 2018; Dunlap 2021b; Garcia-Arias and Schöneberg 2021).

As Tome Valencia Hamilton and Ramcilovic-Suominen (forthcoming in this Special Feature) argue, whether a certain political process or agenda is to result in radical transformations or not will depend not only on their foci and even outcomes but also on the nature of the process of change. Concerning the focus and outcomes, in this paper I have argued for a need of an all-encompassing change and reinvention across all dimensions—from onto-epistemological, to material, to political. The focus should be firmly on justice, race, colonial histories, inequalities, gender, class, and it should ensure that marginalized and colonised voices and concerns are not only attended to but are at the centre of the processes where problems are defined, and solutions designed and implemented. Such focus is a prerequisite not only for co-creating just alternatives and solutions, knowledge, and technologies but also a precondition for safeguarding against co-option by elite interests and actors. Concerning the process of change, as decoloniality, transformations and degrowth imply reshuffling and redistribution of power, wealth and privilege, the process ought to be messy, flexible, deeply democratic and locally owned. Direct democracy, local ownership, local autonomy, collective action and solidarity should be fiercely defended. As the protests for climate justice associated with the COP26 climate change conference in Glasgow bear witness to, it is becoming clear to an increasing number of people that, in the words of the COP26 Coalition for Climate Justice: “The transformative solutions that we need in order to survive and build a more just and fairer world can only be brought about through collective action, solidarity and coordination, from our local communities to international levels” (@COP26_Coalition twitter account, tweet 6.11.2021).

At a more practical level, I wish to highlight the role of social movements and activism, including science and research activism, as a counteraction to socioecologically and socioeconomically harmful and unjust policies and practices (Caniglia et al. 2021; Stephens 2020), but also as a force to help reimagine the ‘ordinary’ and to co-create the ‘alternative’. Scholars, especially those in the Global North, have a moral responsibility to make space for their colleagues from the Global South to tell their truths, as well as to be allies of the marginalized and oppressed societal groups and voices. Building coalitions and synergies between different initiatives and movements for change, which are based on different knowledge and aim to unmake the current structures and status quo together with the underlying logics, are crucial for co-designing alternative ways forward—ways forward where justice, plurality, solidarity, resilience and resistance are both the means and the ends of the processes of change.

## Footnotes

1 In this part the focus is only on the inter-species level, leaving out the dynamics and violence within the human species, such as enslavement, unequal extraction and appropriation of labour and other “resources”, which continue in different forms and with different justifications to the present day.
